# Automatic Segmentation of Ulna and Radius in Forearm Radiographs

**DOI:** 10.1155/2019/6490161

**Published:** 2019-01-29

**Authors:** Xiaofang Gou, Yuming Rao, Xiuxia Feng, Zhaoqiang Yun, Wei Yang

**Affiliations:** ^1^Guangdong Provincial Key Laboratory of Medical Image Processing, School of Biomedical Engineering, Southern Medical University, Guangzhou 510515, China; ^2^Research and Development Department, Shenzhen SONTU Medical Imaging Equipment Co., Ltd, Shenzhen 518118, China

## Abstract

Automatic segmentation of ulna and radius (UR) in forearm radiographs is a necessary step for single X-ray absorptiometry bone mineral density measurement and diagnosis of osteoporosis. Accurate and robust segmentation of UR is difficult, given the variation in forearms between patients and the nonuniformity intensity in forearm radiographs. In this work, we proposed a practical automatic UR segmentation method through the dynamic programming (DP) algorithm to trace UR contours. Four seed points along four UR diaphysis edges are automatically located in the preprocessed radiographs. Then, the minimum cost paths in a cost map are traced from the seed points through the DP algorithm as UR edges and are merged as the UR contours. The proposed method is quantitatively evaluated using 37 forearm radiographs with manual segmentation results, including 22 normal-exposure and 15 low-exposure radiographs. The average Dice similarity coefficient of our method reached 0.945. The average mean absolute distance between the contours extracted by our method and a radiologist is only 5.04 pixels. The segmentation performance of our method between the normal- and low-exposure radiographs was insignificantly different. Our method was also validated on 105 forearm radiographs acquired under various imaging conditions from several hospitals. The results demonstrated that our method was fairly robust for forearm radiographs of various qualities.

## 1. Introduction

Ulna and radius (UR) are sensitive to changes in a human bone mineral density (BMD) index. A low BMD can cause osteoporosis. Osteoporosis is extensively recognized as an important public health problem, given the significant morbidity and mortality associated with its complications, such as fragility fractures. BMD measurements play a crucial role in evaluating patients at risk of osteoporosis [1]. Noninvasive BMD measurement methods mainly consist of single X-ray absorptiometry (SXA), dual-energy X-ray absorptiometry, quantitative ultrasound, and quantitative computed tomography [2]. The SXA methods are extensively applied in clinics, and the methods have the advantages of low radiation, easy operation, and low-cost for patients.

**Figure 1:**
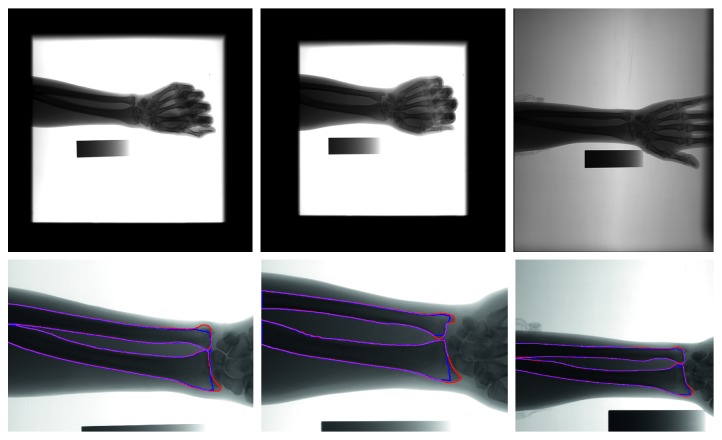
Examples of UR segmentation results through the proposed method. Red and blue contours indicate the UR contours outlined by a radiologist and the automatic segmentation results, respectively. Magenta lines present the overlapping part between the red and blue contours.

Accurate and robust extraction of the UR from forearm radiographs is needed in a BMD measurement system of the SXA. The extraction is a challenging task for several reasons. Large interpatient variations are exhibited in forearm radiographs, as shown in Figure 1. The exposure dose, the directions of forearms, the valid imaging region, and the positions of irrelevant objects vary in the radiographs. The contrast of forearm radiographs is typically low [3]. The contours of the UR in forearm radiographs are weak, fuzzy, and diffusive near the wrist regions that frequently overlap with other bones and soft tissues. In particular, this phenomenon is evident in the forearm radiographs of elderly patients. Intensity inhomogeneity may result in inaccurate pixel classification in several intensity clustering and classification approaches. Furthermore, clinical forearm radiographs have serious global intensity inhomogeneity (Figure 1). The background intensity is uneven globally under the effect of the X-ray tube anode.

A few studies have been conducted on the segmentation of UR in forearm radiographs. Antonio et al. used *k*-means clustering algorithm to semiautomatically segment the distal UR in radiographs [4, 5]. However, the segmentation results are lack of quantitative evaluation in these studies. We had applied *k*-means to segment the forearm radiographs. Nevertheless, it turns out that *k*-means is unsuitable for segmenting our collected forearm radiographs. Pixel-based clustering algorithms may not be robust to noise and serious global intensity inhomogeneous images. Furnstahl et al. applied graph-cuts to segment the UR in 3D forearm computed tomography (CT) images [6]. Furthermore, the statistical pose and shape models [7–10] had been proposed to segment wrist bones in the CT images. The shape model-based methods produce average shapes and are ineffective with abnormal cases. The segmentation performance of the shape models relies on the approximation accuracy of an initial solution. These characteristics have reduced the practicability, robustness, and generalization ability of the methods.

In this work, we aim to develop an accurate and robust method for segmentation of UR in forearm radiographs for practical applications. In contrast to the shape model-based methods, the dynamic programming (DP) algorithm is efficient and simple. Yu et al. used the DP algorithm to effectively trace the optimal seam line between two images [11]. Yang used the DP algorithm to quickly extract the contours of the ribcage field in chest radiographs [12]. Following these studies, we apply the DP algorithm to trace the contours of UR from four seed points in two cost maps. The automatically detected seed points are located in four UR diaphysis and soft tissue edges, correspondingly. Two cost maps are horizontal and vertical. The latter is used to trace the UR diaphysis edges, and the former is used to trace the UR edges near the wrist using the DP algorithm. Three examples of the segmentation results produced by our method are demonstrated in Figure 1.

The remainder of this paper is organized as follows. The framework and details of our method are described in Section 2. The experiments and evaluations are presented in Section 3. The summary is provided in Section 4.

## 2. Methods

### 2.1. Overview

**Figure 2:**
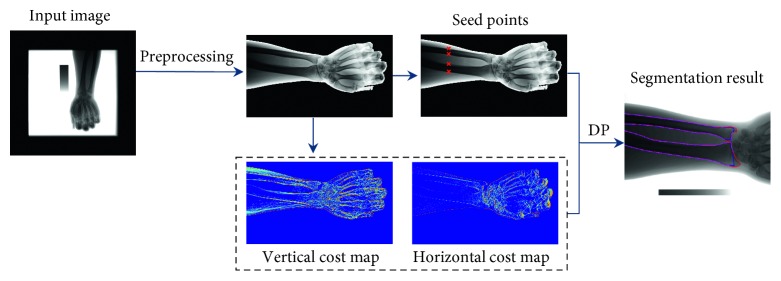
Flowchart of our proposed method for the UR segmentation.

In this work, we aim to develop a practical and robust method for automatic segmentation of UR in forearm radiographs. The core of our proposed method is tracing the minimum cost paths from the seed points to the end points in a reasonable cost map. The flowchart is shown in Figure 2. To effectively perform segmentation and robustly process the varied and low-quality images, a series of preprocessing steps is applied to standardize the input radiographs. First, irrelevant regions are cropped, and the cropped image is down-sampled. Second, the hand direction is corrected to the standard direction to eliminate the hand direction variation for further processing. Third, the forearm and hand mask are extracted. The intensities in the masked regions are normalized into the range [0, 1]. Thus, the interference effect of the background intensity nonuniformity and disturbing objects on further seed point detection are eliminated. Bilateral filter [13] is used to reduce noise, and the histogram equalization algorithm are used to enhance the contrast. After the preprocessing, a standard input image is obtained, as displayed in Figure 2. Four points at the edges of UR diaphysis are then detected as the seed points, and two cost maps (vertical and horizontal) are computed on the preprocessed image. Finally, the DP algorithm is applied to trace four UR diaphysis segments from four seed points in the vertical cost map and the two UR segments near the wrist to close the UR contours.

### 2.2. Preprocessing

In our study, the size of forearm radiographs is very large (i.e., approximately 2816 × 2816 pixels). Given the anatomical shape characteristics of the forearm, the interest region for analysis is small in the input forearm radiographs. Irrelevant background areas can complicate the subsequent detection of seed points. We remove the background regions and downsample the radiographs for subsequent processing. In the original forearm radiographs, the direction of the forearm may vary. Thus, we correct the direction of the forearm into the standard direction (from left to right). Then, we extract the forearm mask, followed by contrast enhancement and denoising. The details of the preprocessing procedure are as follows.

**Figure 3:**
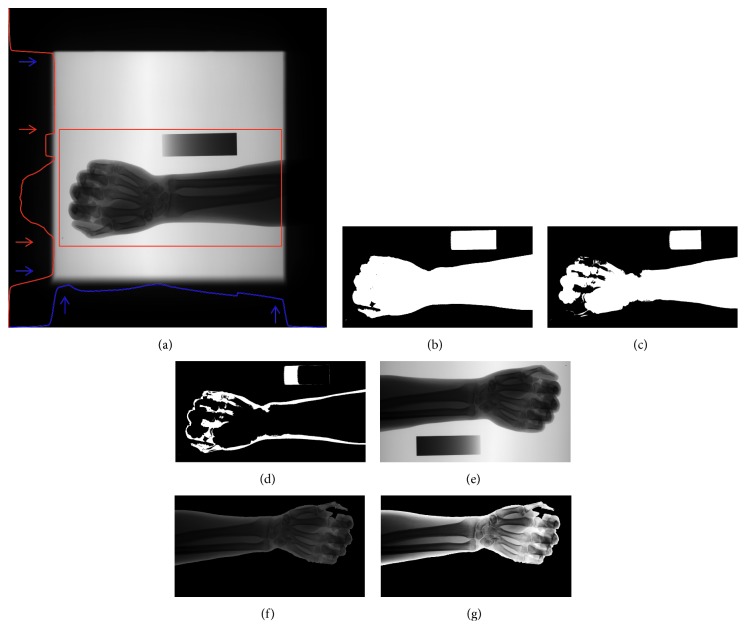
Illustration of preprocessing procedure. (a) Forearm radiographs. (b–d) Binary masks. (e and f) Intermediate results of preprocessing. (g) Final result of preprocessing.

1. Image cropping: first, the invalid regions are cropped in the original forearm radiographs. The cropping coordinate ranges are estimated from the vertical and horizontal intensity projections (Figure 3(a)) of the forearm radiographs. In this figure, the red and blue lines present horizontal and vertical intensity projection lines, respectively. We denote the points (blue and red arrow positions in Figure 3(a)) on the intensity projections as the coordinates for cropping. The red rectangle region displayed in Figure 3(a) is the remaining valid region after removing the invalid regions. Then, the valid region is downsampled by bilinear interpolation.
2. Direction correction: first, the primary direction of the forearm is determined simply by the width and height of the cropped valid region. If the width is smaller than the height of the valid region, the forearm direction is either up or down. In this case, the image is rotated 90° clockwise. Then, the horizontal direction of the forearm is determined by double thresholding the valid region. In Figure 3(d), the binary mask is the difference of the high- and low-threshold binary masks. Figures 3(b) and 3(c) present the binary masks thresholded by the low and high thresholds, correspondingly. If the greatest difference is located in the left of the binary mask, then the forearm direction is from right to left and the valid region is rotated 180° clockwise.
3. Background cleaning: the global thresholding method is used to extract the mask in the valid region of the corrected direction. To assure that the forearm is included in the mask, the forearm mask is enlarged by the morphologic dilation operation. The intensities of the pixels outside of the forearm mask are set to 0, as illustrated in Figure 3(f).
4. Image denoising and contrast enhancement: the bilateral filter is applied to suppress noise in the image obtained in Step (3). Then, the histogram equalization algorithm is applied to enhance the gray contrast, as depicted in Figure 3(g).

### 2.3. Seed Point Detection

**Figure 4:**
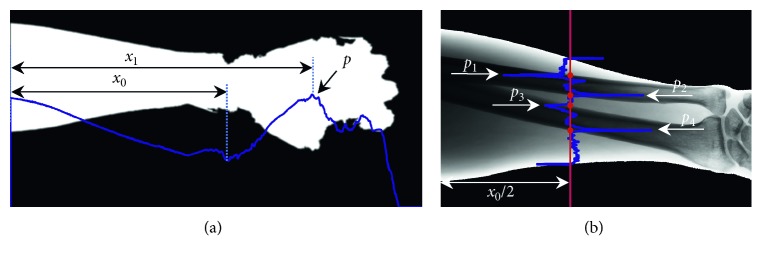
Seed point detection. In (a), the blue line is the vertical intensity projection of the binary mask. In (b), the blue line is the vertical gradient magnitude profile, and *p*_1_, *p*_2_, *p*_3_, and *p*_4_ are the four detected seed points.

To extract the UR contours automatically through the DP algorithm, the seed points on each UR diaphysis and soft tissue edge lines must be identified, respectively. The prior knowledge of the forearm shape and the anatomical structures can help in identifying these seed points. Considering that the four edge lines of the UR diaphysis and soft tissue are more or less horizontally parallel. We use the vertical gradient of a column of the UR to determine the seed points. We locate the horizontal coordinate of the end distal of UR (the *x*_0_ demonstrated in Figure 4(a)). In Figure 4(a), we observe that the end distal of the UR is located at the minimum peak of the vertical intensity projection (the blue line demonstrated in Figure 4(a)). We also limit the search range of the minimum peak from the range of 0 to the maximum peak point *p*.

We observe that the radius, ulna, and skin in the middle of UR are relatively dispersive and easy to distinguish from each other. Thus, we use the vertical gradient profile in the middle of UR to detect the seed points. In Figure 4(b), the blue line presents a vertical gradient profile. The four seed points for tracing the contours of UR diaphysis and soft tissue are located at four strong local maximum peaks along the vertical gradient profile.

### 2.4. Cost Maps

The contours of UR diaphysis are approximately horizontally parallel. By contrast, the wrist joint contours of UR are more or less vertically parallel. The gradient directions of the UR diaphysis and joint edges are different. Two cost maps (*I*_1_ and *I*_2_) are defined using vertical and horizontal gradients for tracing the diaphysis and joint edges near the wrist, correspondingly. We let *I*(*x*, *y*) be the cost value of a point (*x*, *y*) in map *I*. *I*_1_ and *I*_2_ are defined as

(1) $$\begin{array}{c} I_{1}\left(x,\;\;y\right)=\lambda_{1}f_{G_{y}}\left(x,\;\;y\right)+\left(1-\lambda_{1}\right)f_{c}\left(x,\;\;y\right),\\ I_{2}\left(x,\;\;y\right)=\lambda_{2}f_{G_{x}}\left(x,\;\;y\right)+\left(1-\lambda_{2}\right)f_{c}\left(x,\;\;y\right), \end{array}$$

where *f*_*G*_*y*__(*x*, *y*) and *f*_*G*_*x*__(*x*, *y*) are the scaled and inverted vertical and horizontal gradient:

(2) $$\begin{array}{c} f_{G_{y}}\left(x,y\right)=1-\frac{G_{y}\left(x,y\right)}{\max\left(G_{y}\right)},\\ f_{G_{x}}\left(x,y\right)=1-\frac{G_{x}\left(x,y\right)}{\max\left(G_{x}\right)}. \end{array}$$

The second term *f*_*c*_(*x*, *y*) denotes the inverted binary edge mask detected by Canny edge detector:

(3) $$\begin{array}{c} f_{c}\left(x,\;\;y\right)=\left\{\begin{array}{c} 0,\;\text{Canny edge,}\\ 1,\;\text{otherwise.} \end{array}\right. \end{array}$$

Empirically, the weights of *λ*_1_ = 0.3, *λ*_2_ = 0.7 in equation (1) seem to work well in a wide range of images. In this work, the Sobel operators are used to compute the horizontal and vertical gradients. Canny edge is robust to the noise given the nonmaximum suppression and double thresholding procedures. However, the edges detected by the Canny edge detector may not be continuous along the weak edges. The gradients can provide several cues along the weak edges for tracing the contours.

### 2.5. Minimum Cost Path Tracing

The UR contours can be found by searching the minimum cost paths in the cost maps using the DP. Intelligent scissor algorithm uses two-dimensional (2-D) DP to search the minimum cost path between two input interactive points [14]. However, automatic searching UR contours using 2-D DP requires detecting many seed points. By contrast, the priors of the UR shape and orientation benefit for automatic searching UR contours using 1-D DP. In this work, the contours of UR diaphysis and joint near wrist separately are approximately horizontally parallel and vertically parallel in the preprocessed radiograms. Thus, the contours of UR diaphysis/joint near wrist can be traced relatively easy along the horizontal/vertical direction using 1D DP.

**Figure 5:**
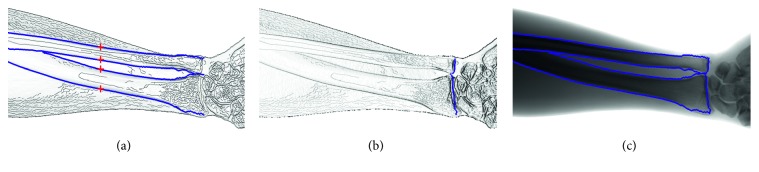
Intermediate results of UR contours tracing. In (a), the blue lines are the traced segments of UR diaphysis in vertical cost map. In (b), the blue lines are the traced segments of UR joint in horizontal cost map. The close UR contours are displayed in (c).

A UR contour can be divided into two UR diaphysis segments and one UR joint segment. First, we search for the diaphysis segments of each UR contour in the cost map *I*_1_ from a seed point located in the middle of the UR. The left part of the UR diaphysis segment is traced from a seed point as the starting point to left. Furthermore, the right part of the UR diaphysis segment is traced from the seed point to the end distal of the UR, as shown in Figure 5(a). Then, the UR joint segment is traced from up to down in the cost map *I*_2_, as shown in Figure 5(b). Two diaphysis segments and the UR joint segment are merged as one close UR contour, as shown in Figure 5(c).

The first step of tracing each sub-UR diaphysis segments is to traverse the vertical cost map from the seed point to the end column and compute the cumulative minimum cost *c*(*i*, *j*) for all possible next column three-connected paths for the seed point (equation (4)). The minimum value of the end column in *c* indicates the end of the minimum cost path. In the second step, we trace back from the minimum end point *c* to find the optimal path location. The end point *c* is set as the seed point of the next sub-UR diaphysis segment. The sub-UR diaphysis segment tracing process is repeated until all UR diaphysis segments are found.

Each UR joint segment is found by tracing the minimum cost path from one radius or ulna diaphysis segment to the next in the horizontal cost map. To reduce computation time and avoid finding unreasonable UR contours, the range of entry (*i*, *j*) is limited to the area from *x*_0_ *−* *n*/10 to *x*_0_ *+* *n*/10 columns in the horizontal cost map of *m* × *n*:

(4) $$\begin{array}{c} c\left(i,\;\;j\right)=\left\{\begin{array}{cc} I\left(i,\;\;j\right)+\text{min}\left(c\left(i,j\pm 1\right),\;\;c\left(i+1,\;\;j\pm 1\right)\right), & i-1<1,\\ I\left(i,\;\;j\right)+\text{min}\left(c\left(i,\;\;j\pm 1\right),\;\;c\left(i-1,\;\;j\pm 1\right)\right), & i+1>N,\\ I\left(i,\;\;j\right)+\text{min}\left(c\left(i-1,\;\;j\pm 1\right),\;\;c\left(i,\;\;j\pm 1\right),\;\;c\left(i+1,j\pm 1\right)\right), & \text{otherwise,} \end{array}\right. \end{array}$$

where *c*(*i*, *j*) presents the minimum sum cost value from point (*i*, *j*) to the seed point. *N* is the row or column number of the image.

## 3. Experiment Results

### 3.1. Experiment Data

We implemented the proposed method in C++ with OpenCV library. The experiments were performed on a PC with 8 GB RAM and an Intel i5-6500 CPU (3.20 GHz). The dataset, which includes 142 forearm radiographs, was collected from different hospitals in Guangdong, China. A total of 37 forearm radiographs outlined by an experienced radiologist were used to evaluate the performance of our method, including 22 normal-exposure radiographs and 15 low-exposure radiographs.

We used the Dice similarity coefficient (DSC) [15], sensitivity (Sens), false positive rate (FPR), mean absolute distance (MAD), and mean signed distance (MSD) as the evaluation metrics. Three parameters, namely, true positive (TP), false positive (FP), and false negative (FN), are required to compute these metrics. DSC, Sens, and FPR were calculated on the basis of the following formula:

(5) $$\begin{array}{c} \text{DSC}=\frac{2\times \left|R_{A}\cap R_{B}\right|}{\left|R_{A}\right|+\left|R_{B}\right|}=\frac{2\text{TP}}{\left(\text{TP}+\text{FN}\right)+\left(\text{TP}+\text{FP}\right)}, \end{array}$$

where |·| is the cardinality of a set. DSC is the overlap ratio between the ground truth mask *R*_*A*_ and the estimated segmentation mask *R*_*B*_. The DSC value close to 1 indicates an improved segmentation:

(6) $$\begin{array}{c} \text{Sens}=\frac{\text{TP}}{\text{TP}+\text{FN}},\\ \text{FPR}=\frac{\text{FP}}{\text{TP}+\text{FN}}. \end{array}$$

A high value of Sens indicates a few leakage segmentation pixels in the segmentation mask. FPR reflects the error ratio of recognizing the background pixels as foreground pixels. A low value of FPR denotes a few error background pixels in the segmentation mask. MAD is calculated to evaluate the difference between the ground truth contour *A* and the automatically extracted contour *B*.

(7) $$\begin{array}{c} \text{MAD}\left(A,B\right)=\frac{1}{\left|S\left(A\right)\right|+\left|S\left(B\right)\right|}\cdot \left(\underset{p\in S\left(A\right)}{\sum }d\left(p,\;S\left(B\right)\right)+\underset{p\in S\left(B\right)}{\sum }d\left(p,\;S\left(A\right)\right)\right), \end{array}$$

where *S*(*A*) and *S*(*B*) represent the pixel sets of *A* and *B*, respectively. *d*(*p*, *S*(*B*)) represents the minimum Euclidean distance from a point *p* to *S*(*B*), and *d*(*p*, *S*(*A*)) represents the minimum Euclidean distance from a point *p* to *S*(*A*). A low value of MAD indicates automatically extracted contours that are close to the ground truth contours:

(8) $$\begin{array}{c} \text{MSD}\left(A,B\right)=\frac{1}{\left|S\left(A\right)\right|+\left|S\left(B\right)\right|}\cdot \left(\underset{p\in S\left(A\right)}{\sum }D\left(p,\;S\left(B\right)\right)-\underset{p\in S\left(B\right)}{\sum }D\left(p,\;S\left(A\right)\right)\right), \end{array}$$

where *D*(*p*, *S*) represents the signed Euclidean distance from a point *p* to a pixel set *S*:

(9) $$\begin{array}{c} D\left(p,S\right)=\left\{\begin{array}{cc} d\left(p,S\right)\text{,} & p\in \Omega_{S},\\ 0\text{,} & p\in S,\\ -d\left(p,S\right)\text{,} & p\notin \Omega_{S}, \end{array}\right. \end{array}$$

where *Ω*_*S*_ is the inside pixel set of contour *S.* A positive value of MSD indicates the ground truth contours are average larger than automatically extracted contours.

### 3.2. Evaluation

**Figure 6:**
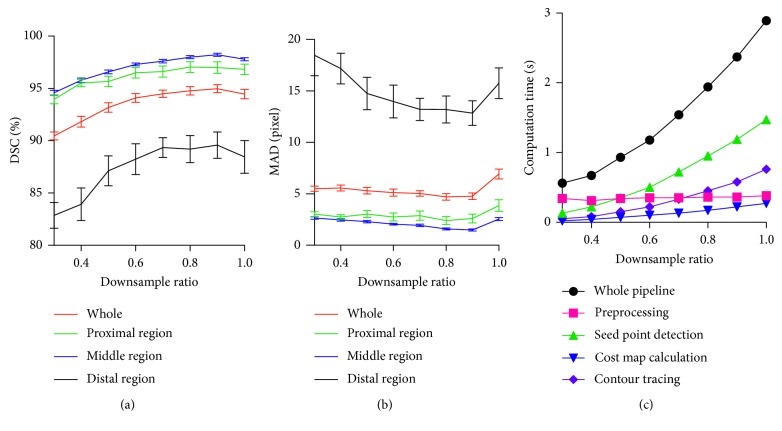
(a) DSC, (b) MAD, and (c) computation time with varying downsample ratios.

We studied the relationship between the segmentation accuracy with the downsample ratios.  Figure 6 exhibits the effects of varying down-sample ratios on the DSC, MAD, and computation time. The proximal, middle, and distal regions sequentially represent the one-third length region of the UR from left to right. We can observe that a stable segmentation performance is achieved with the decrease in the downsample ratio. The computation time approximately linearly decreases with the downsample ratio. When the downsample ratio is smaller than 0.7, the computation time is reduced. However, all four regions of the segmentation evaluation metrics marginally decrease. The majority of the computation time of our segmentation method procedure is consumed during the seed point detection and edge tracing procedures when the downsample ratio is greater than 0.7. The seed point detection and edge tracing time marginally increase with the downscale ratio, as displayed in Figure 6(c). Reliable segmentation results and reasonable performance can be achieved when the downsample ratio is set to 0.7.

**Figure 7:**
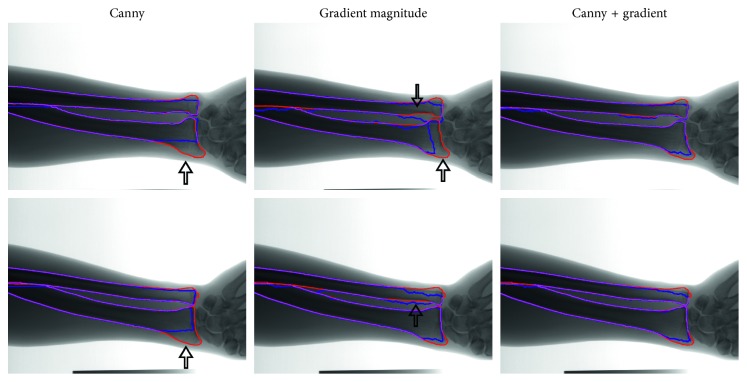
Segmentation results of our proposed method with different cost maps. Red and blue contours indicate the UR contours outlined by a radiologist and the automatic segmentation results, correspondingly. Magenta lines present the overlapping part between the red and blue contours.

In our study, the cost maps significantly affect the segmentation accuracy. An effective cost map must emphasize the object contours. We conducted experiments to evaluate different cost maps and their combinations.  Figure 7 presents the segmentation results obtained through our method with Canny edge, gradient magnitude, and the combined Canny edge and gradient as the cost maps. The results demonstrated that the weighted combination of the gradient with Canny edge as the cost map can lead to more accurate and smooth contours. The cost map with only Canny edge facilitated the poor segmentation, especially near the UR joints, which might be caused by the discontinuous edges and the pseudoedge responses. Furthermore, the cost map with gradient magnitude is sensitive to noise and interference.

**Figure 8:**
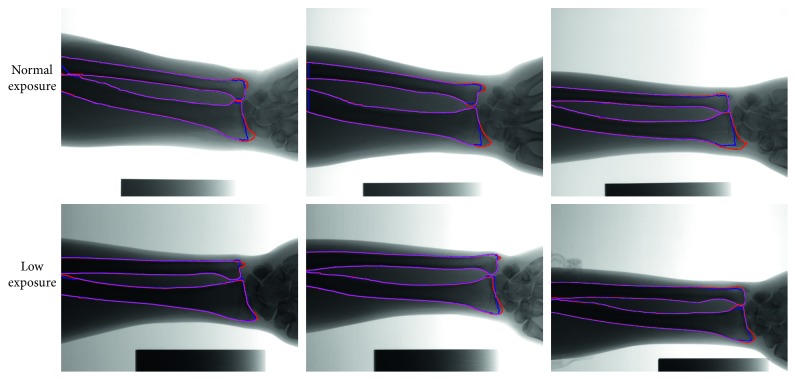
Examples of segmentation results in the normal- and low-exposure forearm radiographs obtained through our proposed method. Red and blue lines indicate manually outlined contours and automatically extracted contours, respectively. Magenta lines represent the overlapping part between the red and blue lines.

We evaluated the robustness of our method for the forearm radiographs of different exposure doses. The examples of the UR segmentation results in the normal- and low-exposure radiographs are illustrated in Figure 8. The automatically extracted contours of all cases are fairly accurate and smooth. The quantitative evaluation metrics of our method on the 22 normal-exposure images are listed in Tables 1–3. The average DSC is 95.02%, the average MAD is only 4.94 pixels, and the average MSD is 3.67 pixels. The variation in quantitative evaluation values among different patients is small. Furthermore, a high segmentation accuracy of our method is achieved on the low-exposure radiographs. The average DSC is 93.69%, the average MAD is only 5.02 pixels, and the average MSD is 3.7 pixels.

The quantitative results on all samples are summarized in Tables 1–3. The average DSC is 94.5%, the average MAD is 5.04 pixels, the average MSD is 3.68 pixels, the average Sens is 90.9%, and the average FPR is less than 1.5%. The segmentation results in the proximal and middle regions are highly accurate for the normal- and low-exposure radiographs. The average Sens in the distal region is 82.64%, and the average FPR is 1.77%. Most segmentation errors have occurred near the wrist joints due to a wide shape variation in these areas.

Our future work aims at improving the cost map and enhancing the difference between the object edges and background edges in the cost map. The cost map of intelligent scissor algorithm [14] is the weighted combination of the Laplacian zero-crossing, gradient magnitude, and gradient direction. The intelligent scissor algorithm is further improved by adding the edge, “inside,” and “outside” pixel values into the cost map in [16]. In [11], the cost map is the weighted combination of intensity similarity, edge similarity, texture similarity, saliency constraint, and location constraint feature map. We also aim at including more effective features into the cost maps. To trace the desired weaker edge nearby strong edge, intelligent scissor algorithm [14] can dynamically update the cost map by on-the-fly training. Training the cost maps for tracing the joint near wrist segments using the already traced object's boundary segments will be considered in the future. And, we intend to detect the corners of the radius and ulna as the additional seed points for tracing the joint near wrist segments. To further correct the traced contours, we will take the construction of a flexible template or model of UR into consideration once we collected enough forearm radiographs with manual segmentation results. Furthermore, the relationship between the accuracy of SXA BMD measurement and the accuracy of segmentation method is under further investigation.

## 4. Conclusions

We presented a practical and efficient method for automatic segmentation of the UR in forearm radiographs. A preprocessing procedure was proposed to standardize the forearm radiographs and make the segmentation method robust to various exposure doses and imaging conditions. The accurate segmentation of our method benefits from the precise detected seed points and two highly discriminative cost maps. Our method is useful for segmenting the UR in forearm radiographs in the SXA BMD measurement system.

## Figures and Tables

**Table 1:** DSC and MAD in the whole, proximal, middle, and distal regions of the UR.

Region	All		Normal exposure		Low exposure	
	DSC (%)	MAD (pixel)	DSC (%)	MAD (pixel)	DSC (%)	MAD (pixel)
Whole	94.49 ± 0.35	5.04 ± 0.28	95.02 ± 0.33	4.94 ± 0.30	93.69 ± 0.69	5.02 ± 0.54
Proximal	96.61 ± 0.54	2.87 ± 0.45	97.54 ± 0.47	2.16 ± 0.43	95.18 ± 1.07	3.97 ± 0.85
Middle	97.61 ± 0.17	1.92 ± 0.13	98.09 ± 0.08	1.60 ± 0.09	95.85 ± 0.34	2.37 ± 0.25
Distal	89.35 ± 0.95	13.21 ± 1.08	89.69 ± 0.82	15.56 ± 1.23	88.82 ± 2.07	9.59 ± 1.58

**Table 2:** MSD in the whole, the proximal, middle, and distal regions of the UR.

Region	All	Normal exposure	Low exposure
	MSD (pixel)	MSD (pixel)	MSD (pixel)
Whole	3.68 ± 0.34	3.67 ± 0.31	3.70 ± 0.71
Proximal	0.72 ± 0.46	0.45 ± 0.46	1.13 ± 0.93
Middle	1.77 ± 0.11	1.49 ± 0.08	2.21 ± 0.22
Distal	10.88 ± 1.11	12.75 ± 1.29	8.01 ± 1.74

**Table 3:** Sens and FPR in the whole, the proximal, middle, and distal regions of the UR.

Region	All		Normal exposure		Low exposure	
	Sens (%)	FPR (%)	Sens (%)	FPR (%)	Sens (%)	FPR (%)
Whole	90.92 ± 0.60	1.45 ± 0.33	91.58 ± 0.53	1.56 ± 0.31	89.91 ± 1.25	1.91 ± 0.70
Proximal	95.82 ± 0.39	2.77 ± 1.42	96.86 ± 0.30	1.82 ± 1.05	94.26 ± 0.71	4.22 ± 2.66
Middle	95.48 ± 0.31	0.14 ± 0.39	96.39 ± 0.15	0.14 ± 0.32	94.07 ± 0.61	0.15 ± 0.08
Distal	82.64 ± 1.49	1.77 ± 0.28	82.81 ± 1.37	1.56 ± 0.29	82.37 ± 3.01	2.11 ± 0.55

## Data Availability

The dataset includes 142 forearm radiographs collected from different hospitals in Guangdong, China. The dataset was collected by Shenzhen SONTU Medical Imaging Equipment Co., Ltd and so cannot be made freely available.
